# Novel Synergies and Isolate Specificities in the Drug Interaction Landscape of Mycobacterium abscessus

**DOI:** 10.1128/aac.00090-23

**Published:** 2023-06-06

**Authors:** Nhi Van, Yonatan N. Degefu, Pathricia A. Leus, Jonah Larkins-Ford, Jacob Klickstein, Florian P. Maurer, David Stone, Husain Poonawala, Cheleste M. Thorpe, Trever C. Smith, Bree B. Aldridge

**Affiliations:** a Department of Molecular Biology and Microbiology, Tufts University School of Medicine, Boston, Massachusetts, USA; b Stuart B. Levy Center for Integrated Management of Antimicrobial Resistance, Boston, Massachusetts, USA; c Graduate School of Biomedical Sciences, Tufts University School of Medicine, Boston, Massachusetts, USA; d Institute of Medical Microbiology, Virology and Hygiene, University Medical Center Hamburg-Eppendorf, Hamburg, Germany; e National and WHO Supranational Reference Center for Mycobacteria, Research Center Borstel, Borstel, Germany; f Division of Geographic Medicine and Infectious Diseases, Department of Medicine, Tufts Medical Center and Tufts University School of Medicine, Boston, Massachusetts, USA; g Department of Biomedical Engineering, Tufts University School of Engineering, Medford, Massachusetts, USA

**Keywords:** NTM, antibiotics, drug interaction, infectious disease, personalized medicine

## Abstract

Mycobacterium abscessus infections are difficult to treat and are often considered untreatable without tissue resection. Due to the intrinsic drug-resistant nature of the bacteria, combination therapy of three or more antibiotics is recommended. A major challenge in treating M. abscessus infections is the absence of a universal combination therapy with satisfying clinical success rates, leaving clinicians to treat infections using antibiotics lacking efficacy data. We systematically measured drug combinations in M. abscessus to establish a resource of drug interaction data and identify patterns of synergy to help design optimized combination therapies. We measured 191 pairwise drug combination effects among 22 antibacterials and identified 71 synergistic pairs, 54 antagonistic pairs, and 66 potentiator-antibiotic pairs. We found that commonly used drug combinations in the clinic, such as azithromycin and amikacin, are antagonistic in the lab reference strain ATCC 19977, whereas novel combinations, such as azithromycin and rifampicin, are synergistic. Another challenge in developing universally effective multidrug therapies for M. abscessus is the significant variation in drug response between isolates. We measured drug interactions in a focused set of 36 drug pairs across a small panel of clinical isolates with rough and smooth morphotypes. We observed strain-dependent drug interactions that cannot be predicted from single-drug susceptibility profiles or known drug mechanisms of action. Our study demonstrates the immense potential to identify synergistic drug combinations in the vast drug combination space and emphasizes the importance of strain-specific combination measurements for designing improved therapeutic interventions.

## INTRODUCTION

Mycobacterium abscessus, a rapidly growing nontuberculous mycobacterium (NTM), is a ubiquitous, opportunistic pathogen commonly found in soil, water systems, and contaminated hospital materials. A steady increase in the morbidity and mortality of pulmonary NTM infections has been reported worldwide, mainly affecting immunocompromised populations and those with preexisting conditions (1–3). Pulmonary M. abscessus infections are notoriously hard to treat due to the plethora of intrinsic mechanisms conferring resistance toward most clinically relevant antimicrobials, including macrolides, aminoglycosides, tetracyclines, and β-lactams (4). In addition to intrinsic resistance, acquired mutational resistance against macrolides and aminoglycosides poses a significant risk for chronically infected patients with increased long-term antibiotic exposure (5). These characteristics contribute to the complex and multifaceted resistome of M. abscessus, creating a major challenge in developing antimicrobial therapies against M. abscessus.

A standard of care to treat M. abscessus has not yet been established (6, 7). Treatment is typically split into an intensive initial phase comprising at least three antibiotics and a continuation phase comprising at least two antibiotics, with duration varying based on factors such as the radiologic extent and susceptibility testing. Treatment of M. abscessus heavily depends on whether the strain harbors inducible or mutational macrolide resistance, emphasizing the prognostic significance of macrolide susceptibility (7). The poor cure rate and the emergence of pan-macrolide and pan-aminoglycoside resistance are indicative of the urgent need to develop more effective therapies for NTM and M. abscessus infections (5). As M. abscessus treatment requires multidrug therapy, systematic interrogation of drug combinations has the potential to identify synergistic combinations that will form the basis for more effective therapies.

Several combination studies for M. abscessus have been performed using traditional checkerboard assays. However, conducting large-scale drug combination measurements is impractical because these assays are too resource intensive. Here, we utilize DiaMOND (diagonal measurement of n-way drug interaction), a measurement and analysis pipeline based on an efficient geometric sampling of a traditional checkerboard assay, to significantly decrease the number of measurements required to generate a systematic, large-scale catalog of drug interactions for M. abscessus as a resource and evaluate drug interaction patterns (8). We also modified the DiaMOND assay to measure the potentiation effects of drugs that are not active but may increase the efficacy of other compounds. Because few antibiotics are active in M. abscessus, potentiator screens may be critical to developing combination therapies in M. abscessus and other NTMs. Our data set includes antibiotics commonly used in the clinic and next-generation antibiotics, such as bedaquiline and the benzimidazole SPR719. We identified many novel synergistic and potentiated drug pairs, suggesting the potential for effective multidrug regimens in the combination drug space. However, drug interactions were highly variable among different clinical isolates, suggesting the need to make isolate-specific drug combination measurements rather than searching for a universal drug combination to treat M. abscessus infection.

## RESULTS

### Design of systematic drug interaction study in ATCC 19977.

To generate a systematic data set of drug interaction profiles comparable to other published drug interaction studies, we measured drug pair responses in the M. abscessus reference strain ATCC 19977, a Mycobacterium abscessus subsp. *abscessus* variant that harbors *erm*(41) (erythromycin ribosomal methylase gene), whose presence is associated with inducible macrolide resistance (9, 10). We measured pairwise combination effects among 22 antibiotics drawn from four categories (Table 1): (i) drugs currently recommended for M. abscessus treatment (amikacin, azithromycin, cefoxitin, clarithromycin, and linezolid) (7), (ii) drugs that spanned a diversity of mechanisms regardless of potency or clinical relevance (amoxicillin, cerulenin, ethambutol, levofloxacin, moxifloxacin, nitrofurantoin, rifabutin, rifampicin, telithromycin, thioridazine, and vancomycin), (iii) drugs in development for M. abscessus infection (bedaquiline and SPR719), and (iv) drugs that show no strong effect on their own but may be used to potentiate other antimicrobials (avibactam, streptomycin, tetracycline, and verapamil).

**TABLE 1:** Summary of antimicrobial agents used in the study and their potencies against M. abscessus ATCC 19977

Antimicrobial agent	General mechanism of action	Abbreviation	IC_50_ (μg/mL)	IC_90_ (μg/mL)
Active drugs				
Amikacin	Protein synthesis	AMK	6.6	110
Amoxicillin	Cell wall	AMX	300	NA^*a*^
Azithromycin	Protein synthesis	AZM	4.3	680
Bedaquiline	Respiration	BDQ	0.16	1.3
Cefoxitin	Cell wall	FOX	9	28
Cerulenin	Cell wall	CER	2.2	7
Clarithromycin	Protein synthesis	CLR	0.45	80
Ethambutol	Cell wall	EMB	31	1,200
Levofloxacin	DNA	LXF	13	200
Linezolid	Protein synthesis	LZD	14	260
Moxifloxacin	DNA	MXF	1.5	35
Nitrofurantoin	DNA	NFT	44	1,800
Rifabutin	RNA	RFB	1.8	23
Rifampicin	RNA	RIF	16	2,100
SPR719	DNA	SPR	0.39	NA^*a*^
Telithromycin	Protein synthesis	TEL	10	NA^*a*^
Thioridazine	Respiration	TZ	23	39
Vancomycin	Cell wall	VAN	9	250
Potentiator candidates				
Avibactam	β-Lactamase inhibitor	AVI	NA^*b*^	NA^*b*^
Streptomycin	Protein synthesis	STR	NA^*b*^	NA^*b*^
Tetracycline	Protein synthesis	TET	NA^*b*^	NA^*b*^
Verapamil	Efflux pump inhibitor	VER	NA^*b*^	NA^*b*^

a These drugs do not inhibit growth by 90% for the range of concentrations that we tested, so they do not have IC_90_s and are listed as not applicable (NA). The maximum tested concentrations for amoxicillin, SPR719, and telithromycin were 678 μg/mL, 4.35 μg/mL, and 15 μg/mL, respectively.

b Potentiator candidates do not inhibit growth by 50%, so they do not have an IC_50_ or IC_90_ and are listed as not applicable (NA).

**FIG 1:**
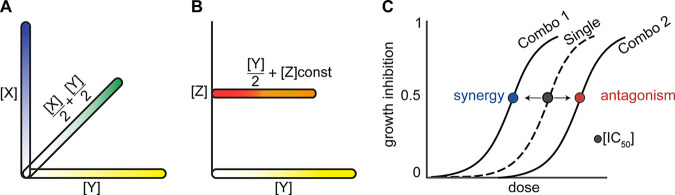
Schematic of drug interaction and potentiation measurement with DiaMOND. (A) Single (axes) and pairwise (diagonal) dose-response sampling using an equipotent mixture of two drugs. The *x* and *y* axes show the doses of the single drugs (X and Y) sampled from low to high concentrations, as indicated by the blue and yellow gradients. The diagonal is the mixture of half of each individual drug at each dose, as shown by the green gradient. (B) Schematic of antibiotic and antibiotic-potentiator dose-response measurement. A dose response for the antibiotic alone (Y; yellow gradient) and with the addition of a constant concentration of the potentiating agent. The antibiotic-potentiator dose response is a mixture of half the dose of the potentiating drug (constant) and half of the nonpotentiating drug (increasing amounts in a dose response), as shown by the red-to-orange gradient. (C) Schematic of shifts in dose-response curves with synergy or antagonism. The dose-response curve in the dashed line represents the effect of a single drug. When the single drug is combined with another drug, the combination curve might shift to the left, indicating synergy (blue dot), or shift to the right, indicating antagonism (red dot).

Drug interactions are calculated by evaluating the combined drug efficacy compared to the single-drug efficacies. As a metric of drug interactions, we calculated fractional inhibitory concentrations at 50% growth inhibition (FIC_50_) based on Loewe additivity and Bliss independence null models. Metrics from both models were strongly correlated (*R*^2^ = 0.75, Pearson correlation; see Fig. S1 in the supplemental material), so we focused on FICs calculated by Loewe additivity. We report log_2_FIC values so that the magnitudes of synergies (negative) are matched to antagonisms (positive). We visualized drug interactions (synergies or antagonisms) by comparing dose-response curves of single and drug combination treatments, as shown in Fig. 1C.

Inherent drug resistance of M. abscessus eliminated several antibiotics from the drug interaction study because synergies are generally evaluated between active compounds (11) (Fig. 1A). Thus, another possibility in developing combination therapy is to include drugs that are inactive alone but potentiate the activity of an active drug. We refer to such candidate compounds as potentiators or attenuators, depending on their effect on partner antibiotics. To quantify the potentiation effect between an active drug and a potentiator candidate, we modified DiaMOND by screening the dose-dependent activity of a potentiator with a constant dose of active drug (dose-responsive) (Fig. 1B). We measured two dose responses: (i) the single-drug dose-response curve with increasing concentrations and (ii) the single-drug dose response (in which drug concentration was reduced by half for all doses) combined with a fixed drug dose (at 1.5× to 2× the reported peak plasma concentrations in humans [Fig. 1B]) (12–15). The effect of the potentiator candidate was calculated as a fold change in concentration of the antibiotic to reach a specific level of growth inhibition (IC_50_ or IC_90_) with the potentiator candidate compared to the drug alone. This fold shift in IC (FsIC) ratio can be interpreted similarly to FIC values: log_2_FsICs are negative for potentiator-drug pairs and positive for attenuator-drug pairs. We focused on evaluating fold shifts at 50% and 90% growth inhibition levels (FsIC_50_ and FsIC_90_, respectively). Potentiation and attenuation can be visualized by the shift of combination dose-response curves relative to single-drug dose-response curves resembling synergy and antagonism, respectively (Fig. 1C), with the exception that the combination dose response utilizes a constant dose of the potentiator candidate with an increasing dose level of the antibiotic.

### A drug interaction landscape of ATCC 19977.

**FIG 2:**
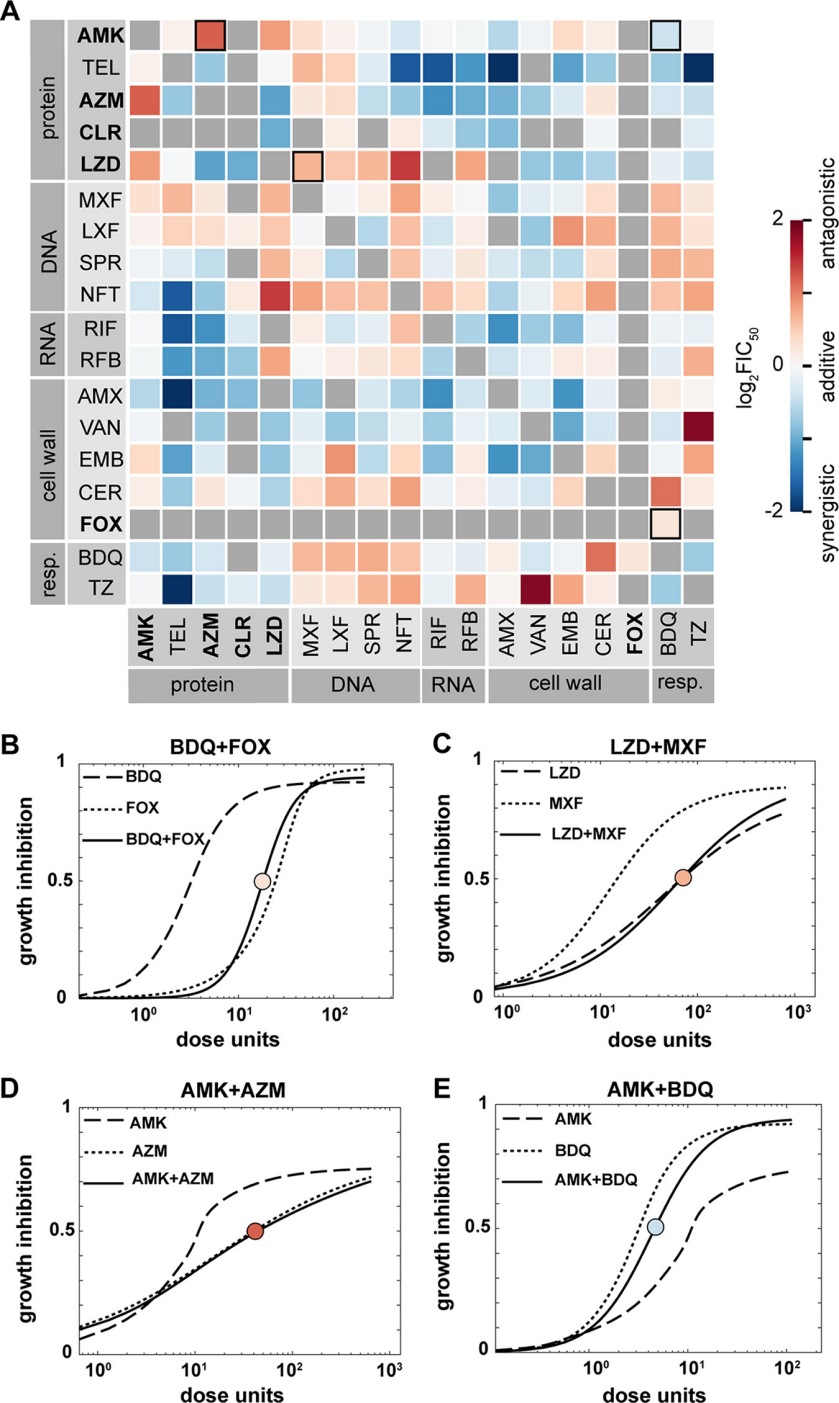
Drug interaction landscape of M. abscessus strain ATCC 19977. (A) Heatmap of pairwise drug interactions among 18 drugs. Drugs are organized by the broad mechanism of action (e.g., inhibitors of respiration [resp.] and the synthesis of protein, cell wall, DNA, or RNA), and drugs recommended for treating M. abscessus infection are indicated in bold text (7). Drug interactions are evaluated with log_2_FIC_50_ values: log_2_FIC of <0 (synergy, blue) and log_2_FIC of >0 (antagonism, red). Gray boxes indicate unmeasurable drug combinations that did not pass quality control. Outlined squares indicate combinations that are shown in the dose responses below. (B to E) Example pairwise (and corresponding single-drug) dose responses. Pairwise dose responses for synergistic and antagonistic combinations are shifted to the left and right, respectively, compared to the expected combination dose-response curves. Circles with the red-blue scale according to log_2_FIC_50_ values as in panel A. The *y* axis represents growth inhibition, whereas the *x* axis represents the dose unit (a unitless representation of the volume, or concentration, used for each drug; a dose unit is preferred when plotting as different drugs have different inhibition concentrations, while a dose unit normalizes the difference and allows easy representation). (B and C) Dose-response curves of combinations that were reported from other studies: bedaquiline (BDQ) + cefoxitin (FOX) (B) and linezolid (LZD) + moxifloxacin (MXF) (C). (D and E) Dose-response curves for the clinically relevant combination amikacin (AMK) + azithromycin (AZM) (D) and the novel combination amikacin (AMK) + bedaquiline (BDQ) (E). Other abbreviations: TEL, telithromycin; CLR, clarithromycin; LXF, levofloxacin; SPR, SPR719; NFT, nitrofurantoin; RIF, rifampicin; RFB, rifabutin; AMX, amoxicillin; VAN, vancomycin; EMB, ethambutol; CER, cerulenin; TZ, thioridazine.

We measured 153 pairwise interactions among 18 drugs representing five drug classes in the reference strain ATCC 19977 (Fig. 2A). Among these combinations, 125 pairs passed our quality control metrics (Materials and Methods). Approximately 20% of the drug interactions were unmeasurable due to extreme variation in drug combination response. We observed similarly high levels of variation in single-drug susceptibilities as those in other reports (16).

We compared our drug interactions with previously reported results to understand how well our data correlated with independent studies. We found a qualitative (synergy versus antagonism) agreement between drug interactions from DiaMOND and other studies. For example, bedaquiline has been shown to eliminate the bactericidal effect of β-lactams (17). We observed a mild antagonism (log_2_FIC_50_ of 0.25) between bedaquiline and cefoxitin, a β-lactam (Fig. 2B), consistent with the prior study. The antagonism can be visually observed by the right shift of the combination dose-response curve (solid line), compared to the expected combination dose-response curve if half-concentrations of bedaquiline and cefoxitin were used together (data not shown). As another example, we observed a mild antagonism between linezolid and moxifloxacin in agreement with interactions reported in the work of Zhang et al. (log_2_FIC_50_ of 0.67 [Fig. 2C]) (18). Combinations such as rifabutin and clarithromycin were reported to be synergistic by Pryjma et al. (19). Using DiaMOND, we also observed that rifabutin and clarithromycin are synergistic (log_2_FIC_50_ of −0.77). Aziz and colleagues suggest that the synergy between rifabutin and clarithromycin is due to the ability of rifabutin to prevent the induction of *erm*(41) (encoding a ribosome methylase) and its transcription factor *whiB7*, thereby suppressing inducible clarithromycin resistance (20). Taken together, we conclude that DiaMOND measurements of M. abscessus drug interactions are comparable to traditional checkerboard approaches used in other studies.

Drug interactions in other bacterial species, including Mycobacterium tuberculosis and Escherichia coli, tend toward antagonism (21, 22). However, of the 125 measurable pairwise drug interactions among 18 drugs in our drug interaction data set, about two-thirds (71 combinations) were synergistic or additive (with near-zero and negative log_2_FIC_50_ values). Therefore, we observed a tendency toward synergy in the landscape of drug interactions in M. abscessus that contrasts with the patterns observed in other bacterial species.

Combination treatment with amikacin and azithromycin (or clarithromycin) is a recommended treatment for macrolide-susceptible M. abscessus (7). We observed a strong antagonistic relationship between amikacin and a commonly used macrolide, azithromycin (Fig. 2D, log_2_FIC_50_ of 1.2). We also tested another macrolide-amikacin pair (clarithromycin-amikacin) but found the combination was unmeasurable because the data fluctuated widely from replicate to replicate. Generally, we did not observe any strong synergistic combinations between amikacin and other tested drugs, except with amoxicillin, bedaquiline, and nitrofurantoin (log_2_FIC_50_ of −0.58, −0.43, and −0.36, respectively). None of these drugs are currently being recommended for use with amikacin (7). The macrolides azithromycin and clarithromycin were more synergistic in combination with other drugs than the aminoglycoside amikacin. Both azithromycin and clarithromycin are strongly synergistic with linezolid (log_2_FIC_50_ of −1.1 and −1.0, respectively), an oxazolidinone that is suggested as an option for M. abscessus treatment (7, 23). Azithromycin and clarithromycin are also synergistic with cell wall-acting drugs except cerulenin, RNA polymerase inhibitors, and respiratory inhibitors (Fig. 2A).

Besides amikacin and macrolides, other drugs recommended for treating M. abscessus are β-lactams (imipenem or cefoxitin), tigecycline, clofazimine, and linezolid (7). Linezolid is strongly antagonistic with the DNA-targeting antibiotics tested here and synergistic with cell wall-acting drugs or respiratory inhibitors (Fig. 2A). Our observations of drug interactions in M. abscessus with multiple compounds targeting the same cellular structure or process (cell wall or cellular respiration/DNA replication, respectively) suggest that the effects of linezolid can be enhanced or abrogated when combined with compounds targeting these general cellular processes. We did not observe a synergistic relationship between linezolid and amikacin, which was previously reported in another study (18). However, several differences between the two studies may account for these discrepancies, including the medium composition (7H9 medium with supplements versus cation-adjusted Mueller-Hinton broth) and strains tested (ATCC 19977 versus clinical isolates from patients). Cefoxitin is one of the few β-lactams recommended to treat M. abscessus besides imipenem (7, 24). We could not obtain a reliable dose-response curve for imipenem and cefoxitin, which may be due to the half-lives of β-lactams in growth medium during the assay (25) or the presence of the *bla_mab_* gene that confers resistance to β-lactams (26). One combination that passed our quality control is bedaquiline and cefoxitin, and it is mildly antagonistic (log_2_FIC_50_ of 0.25). This antagonism may be explained by a previously described mechanism of bedaquiline, wherein it prevents a toxic ATP burst in M. abscessus cells responding to cell wall damage caused by cefoxitin (17, 27).

Another drug candidate for the combination treatment of M. abscessus is bedaquiline, a diarylquinoline that inhibits subunit c of mycobacterial ATP synthase (28). A clinical study demonstrated the efficacy of using bedaquiline as salvage therapy, reducing the bacterial load in the sputum of patients within 3 months (29). Consistent with this finding, we found bedaquiline to be effective as a single agent with a low IC_50_ value of 0.16 μg/mL (Table 1). There was an overall trend of bedaquiline being antagonistic with DNA-acting antibiotics (Fig. 2A). In contrast, bedaquiline is synergistic with protein synthesis-targeting drugs (Fig. 2A). Bedaquiline’s synergy with clinically favored protein synthesis inhibitors, such as amikacin and azithromycin *in vitro* (log_2_FIC_50_ of −0.43 and −0.36, respectively), has not been previously reported to our knowledge. However, positive clinical outcomes following the administration of bedaquiline following amikacin have been reported for macrolide-resistant Mycobacterium fortuitum complex soft tissue infection (30), and another clinical report demonstrated modest favorable clinical outcomes using bedaquiline against M. abscessus (29).

Another candidate for M. abscessus multidrug regimen development is SPR719. SPR720 is a prodrug of SPR719 that targets DNA gyrase (GyrB) and is in phase II clinical development as a new oral agent for NTM pulmonary diseases (16, 31, 32). SPR719 is highly potent, and yet the interaction profile of SPR719 is relatively mild, with a balanced profile of synergies and antagonisms (an even number of synergistic and antagonistic combinations [Fig. 2A]). SPR719 is additive with amikacin (log_2_FIC_50_ of −0.09) and synergistic with drugs from a broad range of classes used to treat NTMs and tuberculosis, including azithromycin and rifampicin (log_2_FIC_50_ of −0.49, and −0.19, respectively), and all cell wall-acting antibiotics tested except cerulenin (Fig. 2A).

We also evaluated the unexplored combination space by analyzing drug interactions among antibacterials not traditionally used to treat NTMs and tuberculosis. One example is telithromycin, a newer generation of macrolide (ketolide) with a slightly different mechanism of action (33, 34). This semisynthetic macrolide binds to ribosomes with higher affinities than those of traditional macrolides and has a clinical advantage over traditional macrolides for bacterial strains with an inducible *erm* gene (34–36). Despite being designed as an alternative for some macrolide-resistant bacteria, telithromycin is not often used in the clinic due to its toxic side effects on the liver (37). We observed particularly strong synergies between telithromycin and azithromycin, SPR719, nitrofurantoin (log_2_FIC_50_ of −0.76, −0.26, and −1.7, respectively), RNA polymerase inhibitors, and respiratory inhibitors (Fig. 2A). We also found that telithromycin is mildly antagonistic with amikacin and additive with linezolid (log_2_FIC_50_ of 0.10 and −0.02, respectively).

### Antibiotic potentiation in M. abscessus.

**FIG 3:**
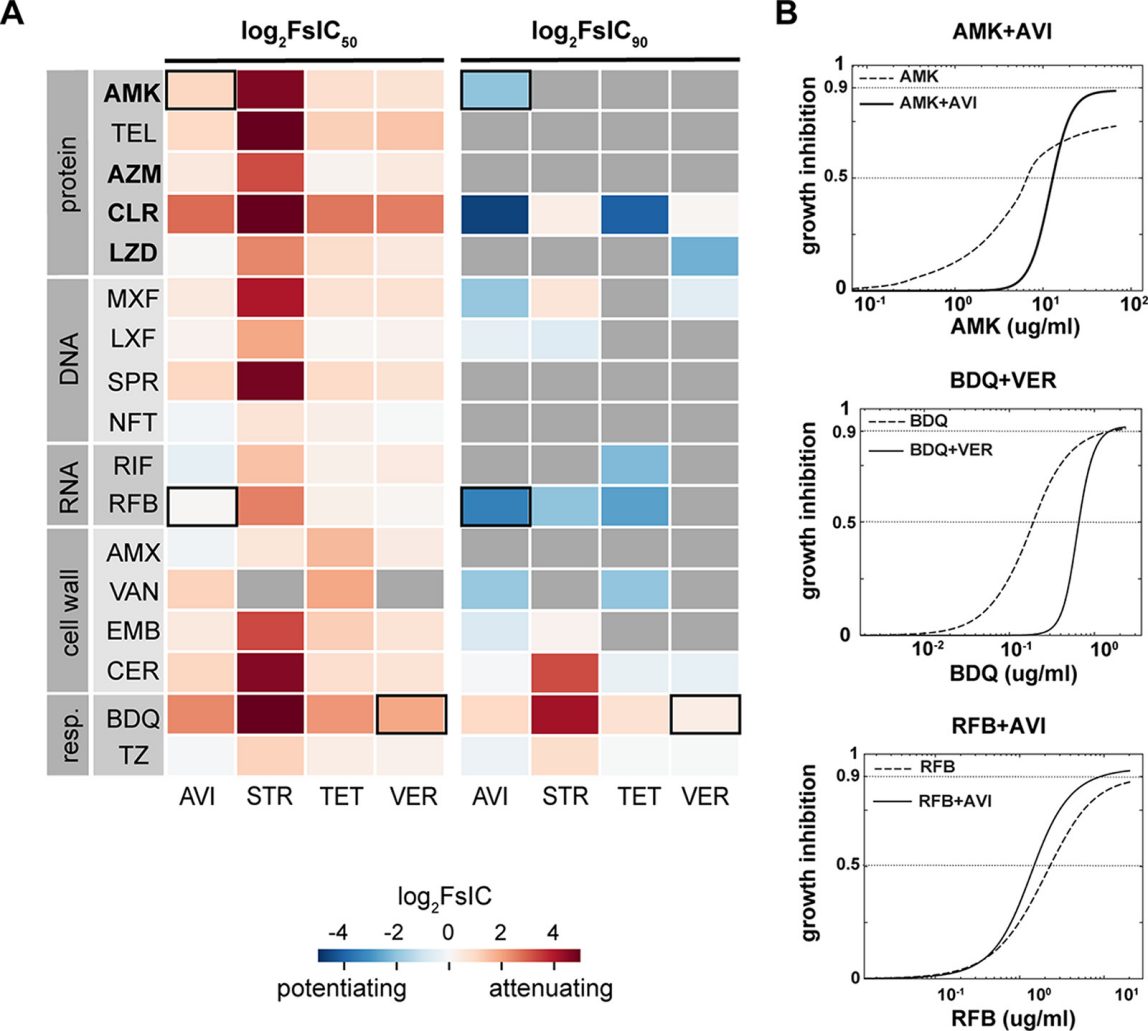
Effect of potentiator candidates on antibiotic efficacies in M. abscessus ATCC 19977. (A) Heatmap of drug efficacy shifts with potentiator candidates at a fixed concentration. Drugs are categorized based on their broad mechanisms of action, and drugs recommended for treating M. abscessus infection are indicated in bold text (7). Drug interaction measurement is expressed as the change in log_2_ fold shift at IC_50_ (left) or IC_90_ (right). A log_2_FsIC of <0 indicates potentiating effects (blue), and a log_2_FsIC of >0 indicates attenuating effects (red). Outlined squares indicate combinations that are shown in panel B. (B) Example dose-response curves showing the fold shift in IC_50_ and IC_90_ for drugs combined with potentiators, amikacin (AMK) + avibactam (AVI) (top), bedaquiline (BDQ) + verapamil (VER) (middle), and rifabutin (RFB) + avibactam (AVI) (bottom). A left or right shift in the combination (solid line) dose-response curve indicates a potentiating or attenuating effect by the potentiator candidate, respectively.

To determine whether a potentiator candidate enhances or attenuates the potency of antibiotics, we measured how the addition of potentiator candidates shifts the partner antibiotic’s dose-response curve (Fig. 1B and C and Fig. 3A). We quantified the degree of potentiation (and attenuation) by calculating the log_2_-transformed fold shift in IC_50_ or IC_90_ of the effective antibiotic with the addition of a constant dose of the candidate potentiator so that a negative log_2_FsIC indicates potentiation and a positive value indicates attenuation (Fig. 1B and C). We observed that none of the four tested potentiator candidates strongly potentiated our antibiotic set at IC_50_; however, some partner antibiotics were potentiated at IC_90_ (Fig. 3A). For example, avibactam attenuates the effect of amikacin at IC_50_ (indicated by the right shift in combination dose-response curves compared to the amikacin dose-response curve; log_2_FsIC_50_ of 1.1 [Fig. 3B]), and yet amikacin reached IC_90_ at a lower concentration with avibactam (log_2_FsIC_90_ of −2.1 [Fig. 3B]), indicating a potentiating effect. We observe a similar shift in all combinations where IC_90_ is reported, e.g., potentiator candidates shifted from attenuating to less attenuating (such as bedaquiline with streptomycin; log_2_FsIC shift from 6.4 at IC_50_ to 4.2 at IC_90_), attenuating to potentiating (as with the previous example between amikacin and avibactam), or less potentiating to more potentiating (such as avibactam and rifabutin; log_2_FsIC shift from 0.04 at IC_50_ to −3.4 at IC_90_ [Fig. 3B]). This dramatic shift from attenuation to potentiation is explained by an increase in the steepness of the dose-response curve with the addition of potentiator candidates so that the effect is an increase in potency at higher dose levels of the antibiotic (Fig. 3B). Among the four tested potentiator candidates, streptomycin has the strongest attenuating effect at IC_50_ and IC_90_ (except with rifabutin and levofloxacin, in which case streptomycin acts as a potentiator) (Fig. 3A).

Due to the presence of the broad-spectrum β-lactamase Bla_Mab_, imipenem and cefoxitin are the only β-lactams currently recommended in multidrug-regimens targeting M. abscessus (5). Therefore, potentiators such as avibactam, a non-β-lactam β-lactamase inhibitor shown to efficiently inhibit Bla_Mab_, can potentially help extend the spectrum of β-lactam antibiotics active against M. abscessus (38). Avibactam has been previously reported to reduce the MIC of cell wall-acting agents (39). Here, we observe a potentiating effect of avibactam with cell wall-acting agents ethambutol, cerulenin, and vancomycin (log_2_FsIC_90_ of −0.76, −0.072, and −1.9, respectively) at IC_90_. Because avibactam is a non-β-lactam β-lactamase inhibitor, we also wanted to determine its effect with amoxicillin, a β-lactam. Due to the low potency of amoxicillin, there is no IC_90_ for amoxicillin. However, at IC_50_, we observed a mild potentiating effect between avibactam and amoxicillin (log_2_FsIC_50_ of −0.2), whereas most antibiotics were attenuated when combined with avibactam (the other combinations are avibactam and nitrofurantoin [log_2_FsIC_50_ of −0.2], avibactam and rifampicin [log_2_FsIC_50_ of −0.44], and avibactam with thioridazine [log_2_FsIC_50_ of −0.061]).

### Drug interactions cannot be predicted from single-drug potencies or broad mechanisms of action.

**FIG 4:**
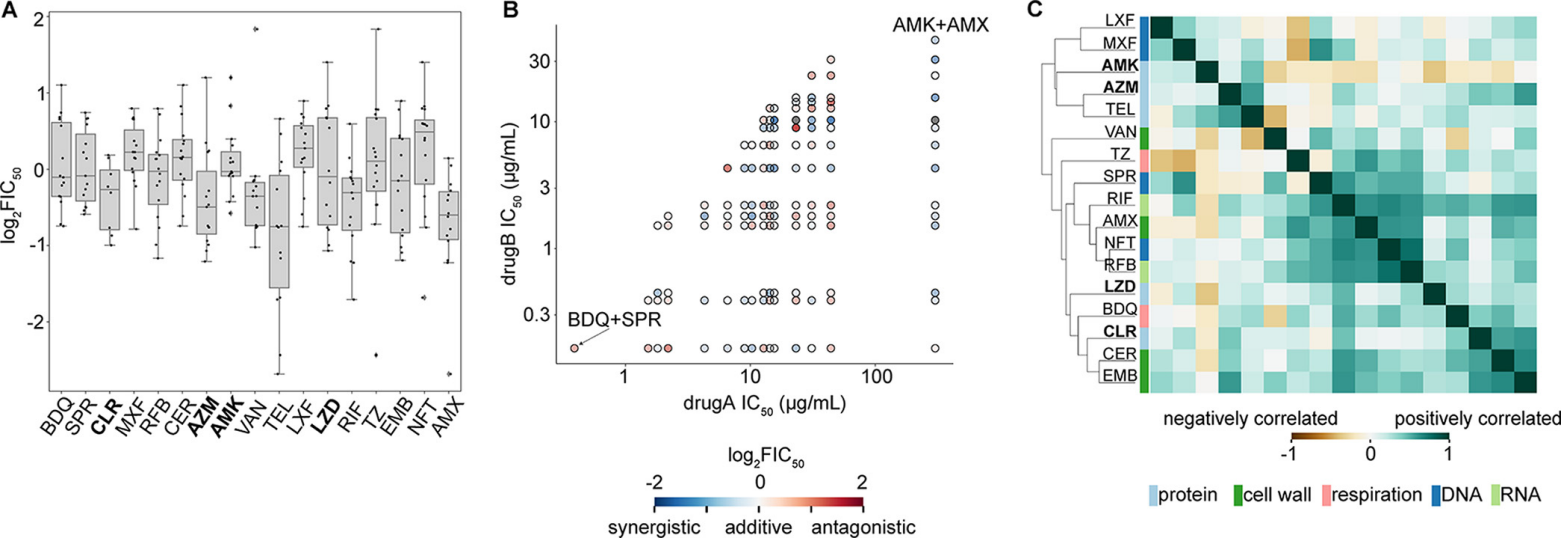
Relationship between drug potency or mechanism of action with synergy. (A) Box plot of drug interactions (log_2_FIC_50_) for all combinations containing the single drug represented on the *x* axis; drugs recommended for treating M. abscessus infection are indicated in bold text (7). Each dot inside the box represents a combination of that single drug and another drug in the data set. Single drugs are ordered on the *x* axis based on IC_50_ (Table 1), with drugs having the highest IC_50_ on the right and those having the lowest IC_50_ on the left. (B) Comparison of drug interaction scores with single-drug potency in ATCC 1977. Pairwise combinations are plotted as the components of their single-drug IC_50_ (micrograms/milliliter). Given a pairwise combination, the potency of a single drug is plotted on the *y* axis while the less potent drug is on the *x* axis; drugs recommended for treating M. abscessus infection are indicated in bold text (7). The color fill indicates the synergistic (blue) or antagonistic (red) interactions in ATCC 19977. (C) Clustering of drugs’ interaction profile in ATCC 19977. Each box represents the Pearson correlation of drug interaction profiles between two antibiotics. The color bar along the *y* axis represents the broad mechanism of action (e.g., inhibitors of respiration and the synthesis of protein, cell wall, DNA, or RNA).

Without a known effective multidrug therapy for M. abscessus infection, clinicians usually rely on single-drug susceptibility profiles and their own experience to determine which drugs should be combined for each patient (7). In some cancers, single-drug susceptibility profiles can be used to design optimized combination therapies under the principle that efficacy for each cancer is determined by its susceptibility to any of the agents and that combination therapies are effective as bet-hedging strategies (40). To understand whether we could predict drug interactions based on single-drug properties, we started by evaluating whether single-drug potencies were correlated with the propensity for drug interactions to be synergistic or antagonistic in M. abscessus. In Fig. 4A, single drugs are organized based on their IC_50_ on the *x* axis, and the *y* axis shows the distribution of log_2_FIC_50_s of all combinations containing that single drug. We do not observe a correlation between distributions of log_2_FIC_50_s and those of IC_50_ for each drug, suggesting that drug interactions cannot be predicted from single-drug potencies. We wondered if the most potent or least potent antibiotic is the driver of drug interaction, which may be obscured by looking at the overall propensity for synergy compared to single-drug potencies. To take the IC_50_ of both drugs in each pairwise combination into consideration, we evaluated whether there were patterns of synergy and antagonism compared to the IC_50_s of both partner drugs (Fig. 4B). In the drug interactions among 18 antibiotics, there was no clear trend of synergy or antagonism based on single-drug potencies (e.g., the drug interactions, colored by log_2_FIC_50_, are not clustered by drug potency to either drug [Fig. 4B]). For example, SPR719 and bedaquiline are highly potent drugs with low IC_50_s (IC_50_s of 0.39 μg/mL and 0.16 μg/mL, respectively), but the combination between SPR719 and bedaquiline is antagonistic (log_2_FIC_50_ of 0.74). Conversely, amikacin and amoxicillin are not as potent as SPR719 or bedaquiline (IC_50_s of 6.6 μg/mL and 300 μg/mL, respectively) but are mildly synergistic in combination (log_2_FIC_50_ of −0.58). Together, our analysis suggests that we cannot anticipate whether a drug pair will be synergistic or antagonistic based on their potency profiles as single agents.

To test the hypothesis that drugs targeting similar pathways would have similar interaction profiles, we compared the similarities among drug interaction profiles from the 17 antibiotics that target four general processes by using hierarchical clustering (Fig. 4C). Cefoxitin is removed from this analysis because there is only one reportable combination containing cefoxitin in our data set. If the drug mechanism of action is a driving factor for drug interactions, we would expect the interaction profiles of similar drugs to be highly correlated. We performed hierarchal clustering on the interaction profiles of each drug and found little clustering based on broad mechanism of action except for two DNA synthesis inhibitors (moxifloxacin and levofloxacin), two cell wall synthesis inhibitors (cerulenin and ethambutol), and two protein synthesis inhibitors (azithromycin and telithromycin) (Fig. 4C). Although we conclude that drug interaction profiles cannot be predicted based on broad mechanism of action, it is possible that an analysis of drug interaction profiles across a larger set of drugs may reveal patterns that are not present in this data set.

### Drug interaction is strain specific.

An extensive study of 85 clinical isolates from M. abscessus subspecies demonstrated species-specific drug susceptibility, including that to clinically favored drugs, such as amikacin and clarithromycin (41). For this reason, we speculated that drug interactions would also vary from isolate to isolate. To test this hypothesis, we selected three clinical isolates (TMC1, TMC2, and TMC3) that differ from each other in their colony morphologies (e.g., smooth versus rough phenotype) and growth rates (e.g., slow versus fast growing). The fastest- and the slowest-growing strains were the lab reference strain ATCC 19977 and TMC2 (4-h and 20-h doubling time, respectively [Table 2]). ATCC 19977 and TMC3 shared similar smooth morphologies, whereas TMC1 and TMC2 shared similar rough morphologies (Table 2).

**TABLE 2:** Characterization of lab strain (ATCC 19977) and clinical isolates TMC1, TMC2, and TMC3

Strain	Doubling time (h)	Morphology
ATCC 19977	4	Smooth
TMC 1	12	Rough
TMC 2	20	Rough
TMC 3	8	Smooth

**FIG 5:**
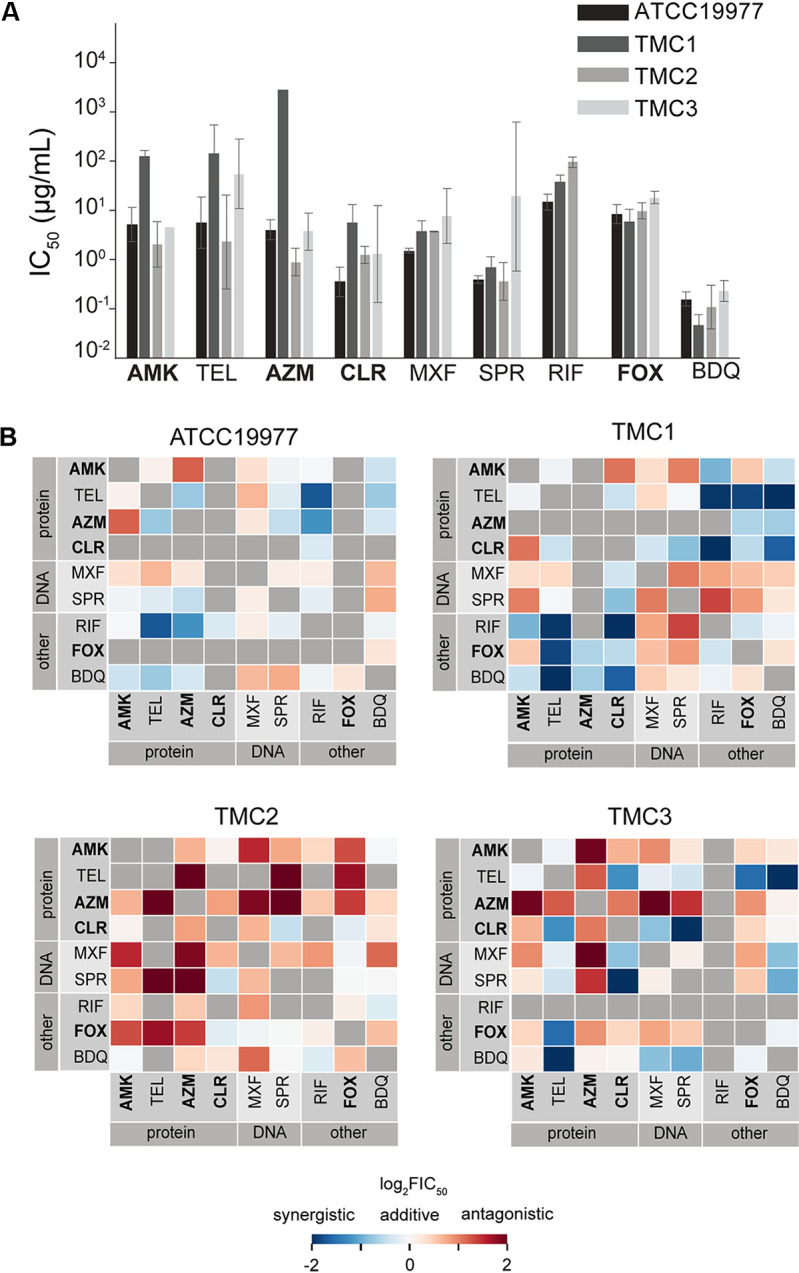
Drug efficacies and interactions in different M. abscessus clinical isolates. (A) Single-drug susceptibilities for the reference strain (ATCC 19977) and three clinical isolates (TMC1, TMC2, and TMC3). Susceptibility is measured by the IC_50_ (micrograms/milliliter). Error bars represent standard deviations. (B) Heatmap showing drug interactions for each strain: ATCC 19977 (top left), TMC1 (top right), TMC2 (bottom left), and TMC3 (bottom right). As in Fig. 2A, drugs are organized by the broad mechanism of action, and drugs recommended for treating M. abscessus infection are indicated in bold text (7). Drug measurement is expressed in log_2_FIC_50_ (color bar).

We used a focused drug set for isolate-to-isolate comparisons; the set includes antibiotics that are recommended for current therapies for NTMs and tuberculosis (amikacin, azithromycin, bedaquiline, cefoxitin, clarithromycin, moxifloxacin, and rifampicin), drugs in development (SPR719), or drugs that are broadly synergistic with other tested drugs in ATCC 19977 (telithromycin). Consistent with previous studies, we observed variation in IC_50_ values across isolates (Fig. 5A) (6, 41). Though susceptibility among strains varies, some relative potencies for single antibiotics are retained across strains. For example, bedaquiline and SPR719 have the highest potencies for the lab reference strain, and this observation largely held for other tested strains (with an exception for SPR719 in the TMC3 strain [Fig. 5A]). To understand if colony morphotype or growth rate was correlated with drug susceptibility, we calculated the Pearson correlation of IC_50_ values between each strain (see Fig. S4A in the supplemental material). In general, we observed poor correlation in single-drug susceptibility patterns between strains, even in strains with common morphologies and similar growth rates (Table 2 and Fig. S4A). The only significant correlation (*R* = 0.88) observed was between TMC2 and ATCC 19977, which differ in morphology and growth rate (Table 2 and Fig. S4A). Together, our data suggest that strain differences in drug response are not well correlated with colony morphology or doubling time.

To understand whether drug interactions were also strain dependent, we systematically measured pairwise drug interactions among the nine drugs in our focused antibiotic set with DiaMOND. The drug interaction profiles varied from isolate to isolate (Fig. 5B). Correlation analysis demonstrates that the most similarity in drug interactions is between TMC1 and ATCC 19977 (*R* = 0.48 [Fig. S4B]). We noted no correlation in single-drug IC_50_ values between TMC1 and ATCC 19977, nor did these two strains share similar morphologies or growth rates. The lack of similarity between these strains, except in the drug interaction profile, supports our hypothesis that drug interaction is independent of single-drug potency and colony morphology. The spectrum of drug interactions is balanced in ATCC 19977, TMC1, and TMC3, whereas TMC2 strongly tends toward antagonism (Fig. 5B). Despite poor overall correlations in drug interaction profiles from strain to strain, we observed some similarities between these strains. For example, amikacin paired with macrolides (such as azithromycin or clarithromycin) is consistently antagonistic. In contrast, combinations of clarithromycin and SPR719 or bedaquiline and telithromycin were all synergistic (Fig. 5B). There are drugs that are largely synergistic, such as clarithromycin with almost all tested drugs for TMC1 (except amikacin) and telithromycin with almost all tested drugs in TMC3 (except azithromycin) (Fig. 5B), suggesting that there may be space for improvement in combination therapy in a strain-specific manner. Taken together, our results affirm the need for targeted drug combination testing and suggest that measurements should be made for each isolate for reliable drug interaction determination.

## DISCUSSION

Infections caused by M. abscessus are notoriously difficult to treat for many reasons, including innate and acquired drug resistance, low antibiotic efficacies, and variation among strains (4, 41). Despite the potential to improve treatment outcomes with combination therapy, we lack a comprehensive drug interaction data set to develop multidrug treatment strategies for M. abscessus infections. In this study, we applied DiaMOND to systematically measure pairwise drug interactions and potentiation among 18 active drugs and 4 potentiator candidates against M. abscessus, resulting in 191 measures in this reference data set.

We have previously found that drug synergies do not necessarily correlate with improved outcomes relative to the standard of care for tuberculosis in mice and clinical trials (21, 42). We have yet to understand the relationship between drug synergy and antagonism for *in vivo* and clinical outcomes for M. abscessus. We expect that once more data are available on treatment outcomes in patients and in animal models, we will be better able to characterize predictive characteristics of *in vitro* synergy and antagonism data with improvements in outcome. The M. abscessus drug interaction data set revealed an unusually large number of synergistic combinations compared to other bacterial species, including M. tuberculosis (21), so we anticipate that translation of drug interaction data in M. abscessus will need to be studied directly and cannot be learned by transferring characteristics from M. tuberculosis and other bacterial pathogens.

Our findings demonstrate that some drugs that are not effective alone potentiate the activities of other antibiotics in M. abscessus. These agents may play an important role in improving M. abscessus regimens, given the poor efficacy of antibiotics as monotherapies for M. abscessus. Among the four candidate potentiators tested, avibactam exhibited the greatest efficacy as a potentiating agent. Surprisingly, we found that avibactam did not enhance the potency of amoxicillin even though combining amoxicillin with avibactam was previously reported to enhance the inhibitory activity of amoxicillin *in vitro* (39). While no conclusions can be made about this discrepancy without further studies, a likely explanation is the differences in experimental procedures and reagents (growth media, strains, temperature, and cellular density) between studies (38, 39).

Some antimicrobials were included in our study to help us understand if drugs that target similar cellular processes elicit similar drug interaction profiles. We found that neither drug potency nor drug mechanism of action can predict drug interaction profiles in M. abscessus. Though drug interaction profiles were not similar among antimicrobials sharing similar broad mechanisms of action, we observed that specific antimicrobials were consistently synergistic or antagonistic with drugs from the same class. For example, the DNA synthesis-targeting compound SPR719 synergized with the peptidoglycan synthesis inhibitors vancomycin and amoxicillin and the arabinogalactan synthesis inhibitor ethambutol. Because peptidoglycan and arabinogalactan are major structural components of the mycobacterial cell wall, the observed synergy in our study may be due to increased accumulation of SPR719 in cells with compromised peptidoglycan or arabinogalactan layers (43–46). A similar trend toward synergy was observed with various representatives of 50S ribosomal subunit inhibitors when combined with rifapentine. Taken together, these results highlight how we may identify synergistic interactions that are dependent on either specific drug classes from systematic data sets, as in the case of vancomycin and amoxicillin combined with SPR719, or specific drug targets, such as the 50S ribosomal subunit inhibitors, which are synergistic with rifapentine. We anticipate that future studies with more antibiotics that have efficacy against M. abscessus, such as carbapenems (47) with SPR719 (48), will provide further insights into synergy patterns and which combinations may be prioritized for studies *in vivo*.

In M. tuberculosis, we have observed that drug interaction measures vary considerably in different growth environments and that we needed to make measurements under multiple conditions that model different aspects of the host environment and make predictive models of *in vivo* outcomes (21). Therefore, we expect that drug regimen development for M. abscessus not only will include measurement of drug combination effects in animal models and clinical trials but also should be expanded to include drug interaction and potentiation experiments performed in several *in vitro* models in parallel. These conditions may include artificial cystic fibrosis sputum and basic growth conditions to model the environment that M. abscessus encounters during infection (21, 49–51). We may also find that other *in vitro* drug response measures, such as CFU assays or RS ratio determination (52), may complement the growth inhibition-based DiaMOND measures to produce *in vitro* data that predict *in vivo* outcomes.

The remarkable variation in drug response among M. abscessus strains, likely due to genomic diversity in NTM species (16, 53), is a major challenge in developing universally effective drug combinations. We measured drug interactions in three clinical isolates with different colony morphologies and growth rates. Previous studies have shown that the impact of colony morphotype, which includes the transition of the smooth colony to the rough colony during infection, influences drug susceptibility (54). We found that drug interactions were poorly correlated across strains and could not be systematically determined based on data from the reference strain. Many other factors can contribute to the variation observed in the data set, such as subspecies and the presence of the *erm* gene (5, 10). Future work focused on measuring drug interactions across a larger set of clinical isolates from known subspecies and genotypes is necessary to address whether a generalized combination therapy could be developed for M. abscessus infections. Nevertheless, our current data suggest that multidrug treatment therapy for M. abscessus may need to be personalized for each isolate (e.g., directly measured despite the challenges in operationalizing isolate-specific testing in the clinic) rather than derived from guidelines based on other strains.

## MATERIALS AND METHODS

### Antimicrobials.

The antimicrobial agents used in this study (except for SPR719) were obtained from Sigma-Aldrich. SPR719 was a gift from Spero Therapeutics. Stock solutions were prepared in dimethyl sulfoxide (DMSO) or sterile water plus 0.01% Triton X-100, depending on solubility, and stored in single-use aliquots at −20°C until used.

### Strains and culturing.

Measurements were made using M. abscessus subsp. *abscessus* strain ATCC 19977 (reference strain) and clinical isolates obtained from patient sputum at the Tufts Medical Center Infectious Disease Clinic (TMC1, TMC2, and TMC3). All strains were cultured in a 7H9 medium supplemented with 0.05% Tween 80, 0.2% glycerol, and 10% BBL Middlebrook albumin-dextrose-catalase (ADC) growth supplement (Middlebrook albumin-dextrose-catalase supplement). Cultures were started from frozen aliquots and allowed to grow to the mid-log phase (optical density at 600 nm [OD_600_] between 0.4 and 0.6), with shaking at 37°C overnight. Cultures were then diluted once to lag phase (OD_600_ between 0.05 and 0.1) and allowed to grow to mid-log phase before performing assays.

### DiaMOND measurement.

DiaMOND was used to measure drug interactions, as previously described for Mycobacterium tuberculosis (8). Details of DiaMOND can be obtained in the work of Van et al. (55). Briefly, DiaMOND uses equipotent drug-combination dose-response curves to approximate the shape of checkerboard isoboles at the same level of growth inhibition. Minor adjustments (e.g., the dose-response curve being centered around 50% inhibitory concentration [IC_50_] in contrast to 90% inhibitory concentration [IC_90_] for M. tuberculosis and the modification of fractional inhibition concentration to fold shifts in inhibition concentration) were made to account for the rapid growth of M. abscessus and the low drug potency relative to M. tuberculosis. Details of these adjustments are explained below.

**(i) Growth inhibition assay.** Assays were performed in clear, flat-bottom 384-well microplates. Drugs were dispensed using a digital drug dispenser (D300e digital dispenser; HP). Drug wells were randomized across plates to minimize plate position effects. Bacterial cultures were diluted to an OD_600_ of 0.05 in fresh medium, and 50 μL of diluted culture was added to each well for drug treatment. Plates were sealed with optically clear plate seals and incubated without shaking at 37°C. Growth (OD_600_) was measured by a microplate reader (BioTek) 48 h after drug treatment.

**(ii) Data analysis.** OD_600_ data were processed using MATLAB’s custom analysis pipeline (The MathWorks). Data were first derandomized from the plate layout, and dose-response curves were organized for each drug and drug combination. The first row of wells in each 384-well plate contained medium-only wells. The median for these medium-only wells was subtracted from each well in a plate as a background. Drug-treated wells were normalized to the mean for the untreated wells (controls), and the values obtained were subtracted from 1 to get a dose-response inhibition curve where 0 and 1 represented no growth inhibition and full growth inhibition, respectively. Growth inhibition curves were then fit to a three-parameter Hill curve using a nonlinear solver in MATLAB (56). The fit accuracy was assessed using the *R*^2^ metric. Equations derived from the Hill function were used to calculate different inhibitory concentration values (IC values) along the dose-response curve.

**(iii) Drug interaction calculation.** To assess whether drugs in combination were synergistic, additive, or antagonistic, we measured the fractional inhibitory concentrations (FICs). We used Loewe additivity as the null model to calculate the FIC values (11). We calculated the expected IC value of the combination AB (drug A with drug B) from the intersection of the combination line to the line of additivity determined by the IC values of A and B alone (Fig. 1A). Finally, the FIC value was calculated by dividing the experimentally observed IC by the expected IC value of the drug combination: FIC = observed IC/expected IC.

We report the log_2_ of the FIC so that the magnitudes of synergy and antagonism scores are balanced around zero. A log_2_FIC value of <0 is synergistic, a log_2_FIC value of 0 is additive, and a log_2_FIC value of >0 is antagonistic. FIC scores were calculated at IC_50_ (FIC_50_) and IC_90_ (FIC_90_). With DiaMOND, we can measure drug interaction scores using other null models such as Bliss independence. By Bliss independence, the expected IC value for [AB] is calculated by multiplying drug A’s effect and drug B’s effect at the desired IC level.

Certain drugs were tested at a fixed concentration (instead of increasing doses) due to their lack of inhibitory effect as a single agent. These drugs are also referred to as potentiator candidates. The concentration of the potentiator candidates was estimated from previously reported serum concentrations (12–15). To quantify drug interaction scores for potentiators, fold shifts in ICs (FsICs) were calculated as a ratio of the observed IC_50_ value of a drug pair combination to the IC_50_ of the active, nonpotentiating drug (Fig. 1B). Drugs with a log_2_FsIC of <0 are potentiated whereas drugs with a log_2_FsIC of >0 are attenuated.

**(iv) Quality control.** Assay quality was assessed in three different ways. The Z-factor was used to determine the quality of the assay at the plate level. For a given plate, the Z-factor was calculated as

$$\text{Z}\prime =1\;-\;\frac{3({\text{σ}}_{\text{positive}}\;+{\text{σ}}_{\text{negative}})}{\left|{\text{μ}}_{\text{positive}}\;-{\text{μ}}_{\text{negative}}\right|}$$

where σ is the standard deviation from the positive and negative control (untreated cells), and μ is the mean from the positive and negative controls. Plates with a Z′ between 0.5 and 1 indicate an excellent assay with a statistically reliable separation between positive and negative controls (57).

The second level of assessment was at the dose-response level. The *R*^2^ value derived from fits of the dose-response curves to Hill curves was used to determine fit accuracy. We combined *R*^2^ values with a visual inspection of the fits. Any Hill curve fits with *R*^2^ values below 0.7 were marked as poor fits and rejected from further analysis. Because of the intrinsic resistance property of M. abscessus, it is challenging to obtain consistent dose-response curves (58). For example, to capture data points close enough to each other to draw an accurate dose response, we need to design doses to increase by 1.5× instead of 2×, which limits the testing range. In addition, the noise of the dose-response curve made fitting challenging. Finally, obtaining a maximum inhibitory concentration for all drugs is difficult due to the heterogeneity of M. abscessus (58). To overcome these difficulties, we designed the experiment around IC_50_ (instead of IC_90_), which is achieved for most drugs and is more reproducible than IC_90_ (Table 1). Lastly, to assess whether doses were sampled in an equipotent manner for combination dose response, the angle of the combination dose response was calculated (the ideal angle is 45°). Angle deviation beyond 22.5° of this ideal equipotent dose response was deemed too far for the approximation of isoboles in the checkerboard and was eliminated from the analysis.

### Clinical isolate growth rate measurement.

To measure the growth rate of clinical isolates obtained from patient sputum at the Tufts Medical Center Infectious Disease Clinic (TMC1, TMC2, and TMC3), suspended cultures of these isolates were grown in a 7H9 medium supplemented with 0.05% Tween 80, 0.2% glycerol, and 10% BBL Middlebrook growth supplement. Cultures were started from frozen aliquots and allowed to grow to the mid-log phase (OD_600_ between 0.4 and 0.6), with shaking at 37°C overnight. Cultures were then diluted once to the lag phase and allowed to grow to the mid-log phase again before performing the assay. Growth rate measurement was performed in a 96-well microplate, with five biological replicates per isolate. Reference strain ATCC 19977 was also included for reference purposes. One hundred fifty microliters of culture at OD_600_ around 0.05 was dispensed into each well. The microplate was sealed with an optically clear plate seal and incubated inside a plate reader at 37°C. OD_600_ was recorded for 18 h at 30-min intervals. Doubling time was calculated using OD_600_ values closest to the log phase. Doubling time was calculated as *r* = ln(OD_600_′ − OD_600_)/(*T*′ − *T*) and doubling time = ln_2_/*r*.
